# Geranylgeranylacetone promotes human osteosarcoma cell apoptosis by inducing the degradation of PRMT1 through the E3 ubiquitin ligase CHIP

**DOI:** 10.1111/jcmm.16725

**Published:** 2021-06-21

**Authors:** Lucen Jiang, Jia Liao, Jianghuan Liu, Qingzhu Wei, Yiyang Wang

**Affiliations:** ^1^ Department of Pathology The Third Affiliated Hospital of Southern Medical University Guangzhou China; ^2^ Department of Pathophysiology School of Medicine Jinan University Guangzhou China

**Keywords:** Apoptosis, CHIP, Geranylgeranylacetone, PRMT1, ubiquitin

## Abstract

Geranylgeranylacetone (GGA), an inducer of heat shock proteins, exerts anticancer activity in some tumours. However, the effect of GGA on human osteosarcoma (OS) has not been reported. This work is designed to evaluate the effect of GGA on the proliferation and apoptosis of human OS cells and to explore the underlying mechanisms. It was found that GGA markedly inhibited the proliferation and induced apoptosis of U‐2 OS cells in a dose‐dependent manner and also up‐regulated the expression of heat shock protein 70 (Hsp70). The degradation and ubiquitination of protein arginine N‐methyltransferase 1 (PRMT1) were obviously enhanced in U‐2 OS cells with CHIP overexpression and GGA treatment. The expression of PRMT1 was reversed in GGA‐treated cell after CHIP knockdown. The turnover of PRMT1 was obviously faster in cells overexpressing CHIP than that in control cells. The methylation and activity of STAT3 were induced by PRMT1, resulting in the inhibition of FAS transcription. Overexpression of PRMT1 reversed the effect of GGA on activation of apoptosis‐related proteins and U‐2 OS cell apoptosis. The expressions of PRMT1 were significantly up‐regulated in OS tissues compared with the adjacent normal tissues and benign bone tumours. In conclusion, GGA promotes the degradation of PRMT1 through the Hsp70‐CHIP‐mediated proteasome pathway, thereby inducing the FAS‐triggered cell apoptosis. Inhibition of PRMT1 may be a potential therapeutic strategy for OS patients.

## 1 INTRODUCTION

Osteosarcoma (OS) is the most common primary skeletal sarcoma that predominantly occurs in adolescents worldwide.^1^, ^2^ Adolescents diagnosed with OS are still treated with chemotherapeutic drug regimens that have remained mostly unmodified since the consolidation of methotrexate, doxorubicin and cisplatin treament in the early 1980s.^2^ The therapeutic outcome is far from satisfactory with a poor median survival time.^3^ Therefore, exploring new therapeutic medicines that effectively block OS progression may provide new strategies for the treatment of OS.

Geranylgeranylacetone (GGA) is an oral anti‐ulcer drug with no major adverse effects.^4^ Previous studies have shown that GGA acts as an inducer of heat shock protein 70 (Hsp70), which rapidly accumulates in cells after heat stress.^5^ At the same time, GGA inhibits cell survival and induces apoptosis in multiple types of cancer, such as leukaemia, colon cancer and melanoma.^7^, ^8^, ^9^ GGA also exerts anti‐invasion effects on ovarian cancer and breast carcinoma cells.^10^, ^11^ Although pro‐apoptotic effects of GGA have been reported, the underlying mechanisms have not been well defined.

The carboxyl terminus of Hsp70‐interacting protein (CHIP), also named STIP1 homology and U‐box‐containing protein 1 (STUB1), is both an Hsp70/Hsp90 co‐chaperone and an E3 ubiquitin ligase.^11^ CHIP performs cellular protein quality control and regulates some important signalling pathways by inducing ubiquitin‐mediated degradation of its substrate proteins via the proteasomal pathway.^12^ The activity of CHIP is initiated through interacting with Hsp70, subsequently promotes protein degradation by polyubiquitination.^13^ Recently, our preliminary data demonstrated that CHIP could promote ubiquitination and degradation of protein arginine N‐methyltransferase 1 (PRMT1) in HEK293 cells.^14^ Other studies have reported that PRMT1 is one of the major protein arginine methyltransferases that catalyses mono‐methylation and asymmetric demethylation of arginine‐bearing substrates.^15^ Thus, PRMT1 is involved in regulating a wide range of cellular processes, including RNA processing, DNA damage response and signal transduction. Aberrant expression of PRMT1 has been found in several malignancies.^16^ Therefore, we speculate that the suppressive effects of GGA on cancer cells are associated with Hsp70‐CHIP‐induced ubiquitination and degradation of PRMT1.

Although there are several reports about the anticancer activity of GGA in tumours, the effect of GGA on human OS has not been evaluated. Therefore, in the present study, the role of GGA in the proliferation and apoptosis of U‐2 OS cell, a human osteosarcoma cell line, was investigated. We also aimed to determine whether the effects of GGA are mediated by CHIP‐induced ubiquitination and degradation of PRMT1.

## 2 METHODS AND MATERIALS

### 2.1 Materials

GGA was purchased from Sigma‐Aldrich (6809‐52‐5, St. Louis, MO, USA). Furamidine dihydrochloride (DB75, HY‐110137) was obtained from MedChemExpress (Monmouth Junction, NJ, USA). Antibodies recognizing Hsp70/Hsc70 (sc‐65521), PRMT1 (sc‐166963), CHIP (sc‐66803), Ubiquitin (sc‐8017), STAT3 (sc‐8019), p‐STAT3(sc‐8059) and Hsp 70 siRNA (sc‐29352) were purchased from Santa Cruz Biotechnology, Inc (Santa Cruz, CA, USA). Anti‐FAS antibody (ab82419) and anti‐mono and dimethyl‐arginine antibody (ab412) were obtained from Abcam (Cambridge, UK). Antibodies against PRMT1 (#2449S), cleaved caspase‐3 (#9664), caspase‐3 (#9662), cleaved caspase‐8 (#9748), caspase‐8 (#4790), cleaved caspase‐9 (#9505), caspase‐9 (#9508), Bax (#14796), Bcl‐2 (#15071), Cyt c (#11940), p53 (#9282), Lamin B (#13435) and GAPDH (#2118) were purchased from Cell Signaling Technology, Inc (Beverly, MA, USA).

### 2.2 Cell proliferation assay

Cell proliferation was detected by Cell Counting Kit‐8 (CCK‐8; CK04‐11, Dojindo, Kumamoto, Japan) assay. U‐2 OS cells were seeded into 96‐well plates at a density of 1 × 10^4^ cells/mL and treated with GGA at various doses for 24 hours. After washing, 10 µl of CCK‐8 was added to each well and incubated for 2 hours at 37℃. The optical density (OD) at 450 nm was detected by Epoch 2 Microplate Spectrophotometer.

### 2.3 EdU staining

Detection of EdU incorporation into the DNA was performed with the Click‐iT EdU Alexa Fluor‐647 Cell Proliferation Kit (C10340, Thermo Fisher Scientific) according to the manufacturer's instructions. In brief, cultured U‐2 OS cells were plated in 24‐well plates at a density of 2x10^5^ cells/well. The cells were treated with GGA for 24 hours before exposure to EdU. After incubation for 2 hours, cells were washed three times with PBS and then fixed with 4% paraformaldehyde for 10 minutes and permeabilized with 0.5% Triton X‐100 for 10 minutes. The cells were washed three times and then incubated with the EdU staining cocktail for 30 minutes protected from light. Images were taken by using fluorescence microscope.

### 2.4 Terminal deoxynucleotidyl transferase dUTP nick end labelling (TUNEL) assay

Cell apoptosis was determined by TUNEL assay (Roche), as previously described.^17^ Briefly, U‐2 OS cells were treated with GGA for 24 hours. After washing with PBS, U‐2 OS cells were fixed with 4% paraformaldehyde and then permeabilized with 0.1% Triton X‐100. The fixed cells were subjected to the TUNEL assay according to the manufacturer's instructions. Finally, the cells were incubated with 4',6‐diamidino‐2‐phenylindole (DAPI) for 15 minutes. Fluorescent images were taken in 10 random fields for each sample using fluorescence microscope.

### 2.5 Western blotting analysis

Western blot experiments were performed as previously described.^17^ Cells were lysed in RIPA buffer. Proteins were separated and transferred to PVDF membranes. After blocking with 5% skim milk, the membranes were incubated with primary and secondary antibodies. The immunoblotted proteins were visualized with enhanced chemiluminescence (ECL) reagents. ImageJ software, an open‐source image processing program, was used to quantify blots.

### 2.6 Co‐immunoprecipitation

The co‐immunoprecipitation assay was performed as described previously.^14^ In brief, cells were treated with 10 μM MG132 for 2 hours and then lysed in RIPA buffer on ice for 30 minutes. Then, the lysates were incubated with anti‐PRMT1 primary antibody and protein A/G‐agarose beads (sc‐2003, Santa Cruz, CA, USA) overnight at 4℃. For dimethyl‐arginine immunoprecipitations, without MG132 treatment, cell lysates were incubated with mono/dimethyl‐arginine antibody and protein A/G‐agarose beads overnight at 4℃. After the final wash, the beads were resuspended in 40 μL 1 × Laemmli buffer. The samples were boiled for 5 minutes and analysed by Western blot probed with the antibody specific for ubiquitin.

### 2.7 Quantitative real‐time polymerase chain reaction (qPCR) analysis

Total RNA was isolated from cells or tissues using TRIzol reagent according to the manufacturer's instructions. RNA samples were reverse‐transcribed using random hexamer primers in the presence of RNase inhibitor (Takara Bio, Shiga, Japan). The cDNA was amplified using the ChemoHS qPCR mix (Monad), 0.2 μM of each primer and nuclease‐free water. Amplified cDNA signals were detected and analysed by CFX Maestro Software v1.1 (Bio‐Rad) using β‐actin as an endogenous control. The results are expressed as the fold increase over control. The specific primers used were listed as follows. FAS: forward 5’‐ GCTGGGCATCTGGACCCTCCTACCT‐3’, reverse 5’‐CAGTCACTTGGGCATTAACACTT‐3’. PRMT1: forward 5’‐AGGCCGCGAACTGCATCATG‐3’, reverse 5’‐GGCCTTGGCAGCAAACATGC‐3’. β‐Actin: forward 5’‐GGCATCCTCACCCTGAAGTA‐3’, reverse 5’‐ AGGTGTGGTGCCAGATTTTC‐3’.

### 2.8 Immunohistochemical analysis

This study was approved by the relevant institutional review board. Whole tissue sections of 39 OS (22 osteoblastic, 9 chondroblastic and 8 fibroblastic) and 41 benign bone tumours (20 osteoid osteomas and 21 chondroblastomas) were evaluated for expression of PRMT1 and Hsp70. In addition, the adjacent normal tissue specimens were served as control. Some paraffin sections were used for haematoxylin and eosin (H&E) staining. Other paraffin‐embedded tumour tissues of patients were cut into 4‐µm‐thick sections. Antibodies against PRMT1 and Hsp70 were used to analyse the sections, respectively. Immunohistochemical staining was performed according to the manufacturer's instructions. Then, the sections were visualized using a microscope (BX45, Olympus Corporation, Tokyo, Japan). PRMT1‐ and Hsp70‐positive cell number and intensity were determined in five randomly selected fields per section at high magnification. Afterwards, the mean of the values from five images per chamber was calculated. The extent of staining was scored according to the percentage of positive cells as follows: 1, <5%; 2, 5%‐25%; 3, 25‐50%; 4, 50‐75%; 5, >75%. The staining intensities (intensity score) were graded as follows: 0, no staining; 1, weak staining; 2, moderate staining; and 3, strong staining. The final score was calculated by the following formula: total score = percentage score × intensity score.

### 2.9 Statistical analysis

Mean densitometry values and all other quantitative data are presented as the mean ± standard error of the mean (SEM). Comparisons among groups were made using Student's *t* test or one‐way ANOVA followed by the Student‐Newman‐Keuls test. Differences with *P* values <0.05 were considered statistically significant. SPSS software was used to analyse the data.

## 3 RESULTS

### 3.1 GGA dose‐dependently inhibited proliferation of U‐2 OS cells and promoted cell apoptosis

**FIGURE 1:**
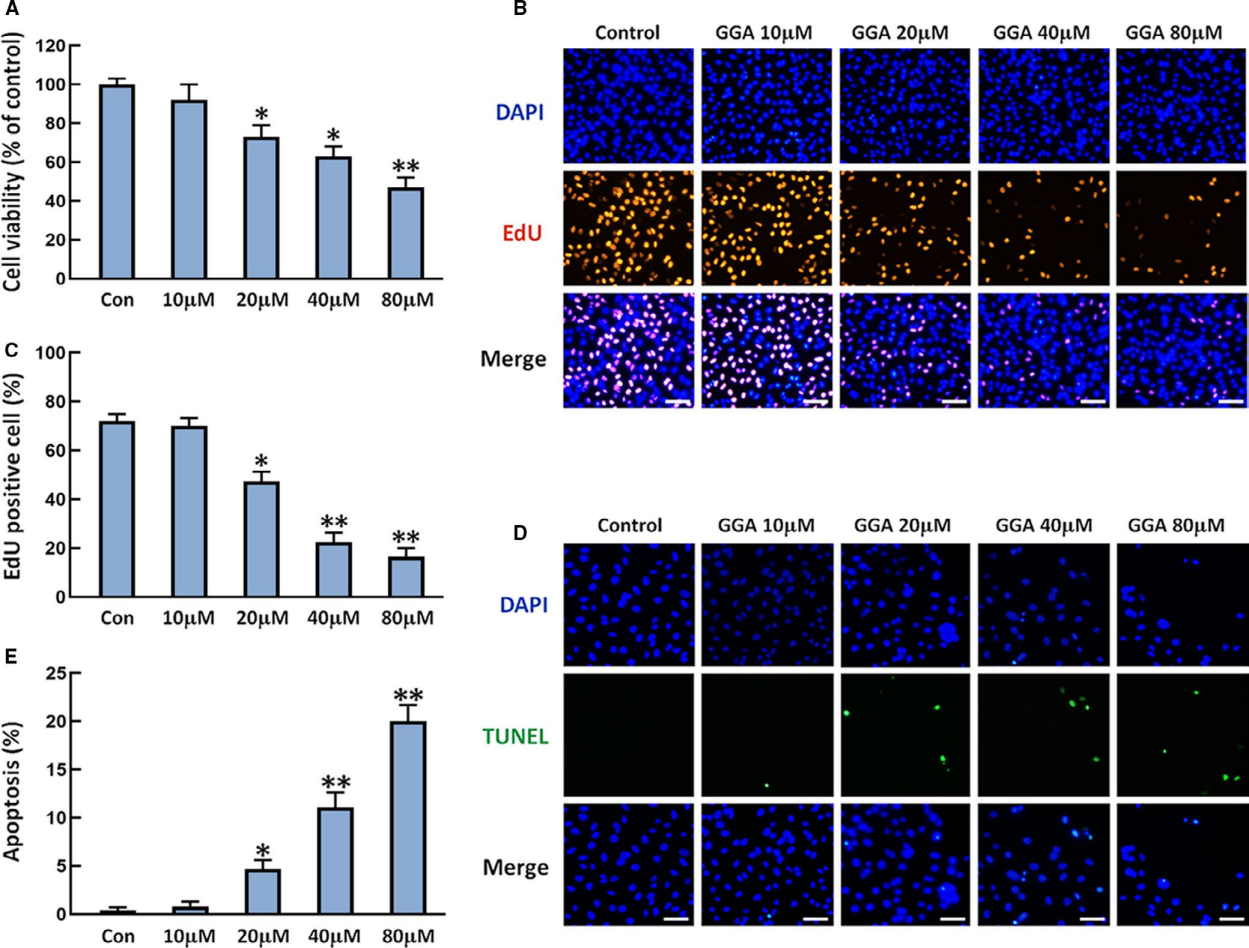
Effects of GGA on the proliferation and apoptosis of U‐2 OS cells. U‐2 OS cells were treated with GGA for 24 h. A, CCK‐8 assay detection of U‐2 OS cell viability. B, EdU staining for cell proliferation. Scale bar = 100 µm. C, Quantification of EdU‐positive cells. D, Representative images of TUNEL assay (green) and total nuclear staining with DAPI (blue). Scale bar = 100 µm. E, the numbers of apoptotic cells were counted in five randomly selected fields for each sample based on the TUNEL images. n = 3. *P < 0.05 and **P < 0.01 vs the control (Con) group

We investigated whether GGA influenced the viability and proliferation of U‐2 OS cells in vitro using the CCK‐8 assay and EdU staining. As shown in Figure 1A, the proliferative activity of U‐2 OS cells was inhibited dose‐dependently in the presence of GGA. The proliferation of U‐2 OS cells was markedly reduced by GGA treatment in a dose‐dependent manner (Figure 1B and C). To assess the direct effect of GGA on apoptosis in U‐2 OS cells, we performed the TUNEL assay in cells treated with various concentrations of GGA for 24 hours. GGA induced U‐2 OS cell apoptosis in a dose‐dependent manner. The apoptosis was markedly increased in U‐2 OS cells after GGA treatment at concentrations above 20 µM (Figure 1D and E). Therefore, we selected 20 µM of GGA for subsequent experiments.

### 3.2 GGA increased tumour necrosis factor receptor superfamily member 6 (FAS) expression and activation of caspase‐3, caspase‐8 and caspase‐9, but did not affect p53 level in U‐2 OS cell

**FIGURE 2:**
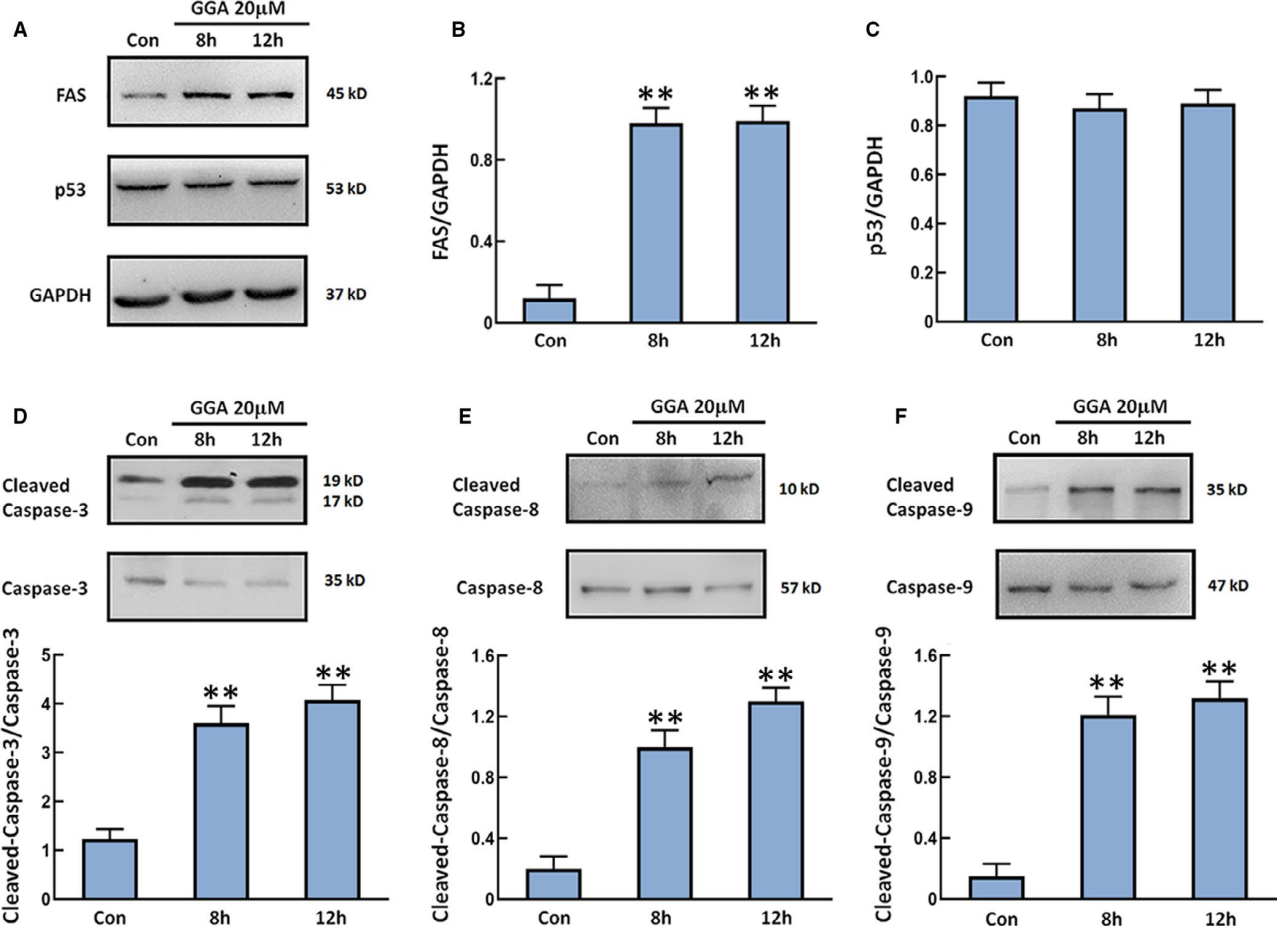
The expression of FAS and p53 and activation of caspase‐3, caspase‐8, caspase‐9 in cells. U‐2 OS cells were treated with 20 µM GGA for 8 and 12 h followed by lysis in RIPA buffer. A, Western blot detection of the expression of FAS and p53. B and C, Quantification of the expression of FAS and p53. D‐F, The levels of cleaved caspase‐3/caspase‐3, cleaved caspase‐8/caspase‐8 and cleaved caspase‐9/caspase‐9 were examined by Western blot and plotted in the panels under the images. n = 3. **P < 0.01 vs the Con group

FAS‐ and p53‐mediated signalling pathways play important roles in the process of apoptosis.^18^ We detected the levels of FAS and p53 in U‐2 OS cells treated with 20 µM GGA at different time points. As shown in Figure 2A‐C, the expressions of FAS were markedly increased in U‐2 OS cells after GGA stimulation for 8 and 12 hours. There was no obvious difference in p53 expression in U‐2 OS cells with or without treatment of GGA. The levels of cleaved caspase‐3, caspase‐8 and caspase‐9 were higher in U‐2 OS cells treated with GGA than those in control cells (Figure 2D‐F). These findings indicate that the suppressive effects of GGA on U‐2 OS cells are closely associated with the activation of the FAS‐mediated apoptotic pathway but not with the p53 pathway.

### 3.3 The level of PRMT1 was decreased in GGA‐treated U‐2 OS cells with concomitantly enhanced PRMT1 polyubiquitination that was regulated by Hsp70

**FIGURE 3:**
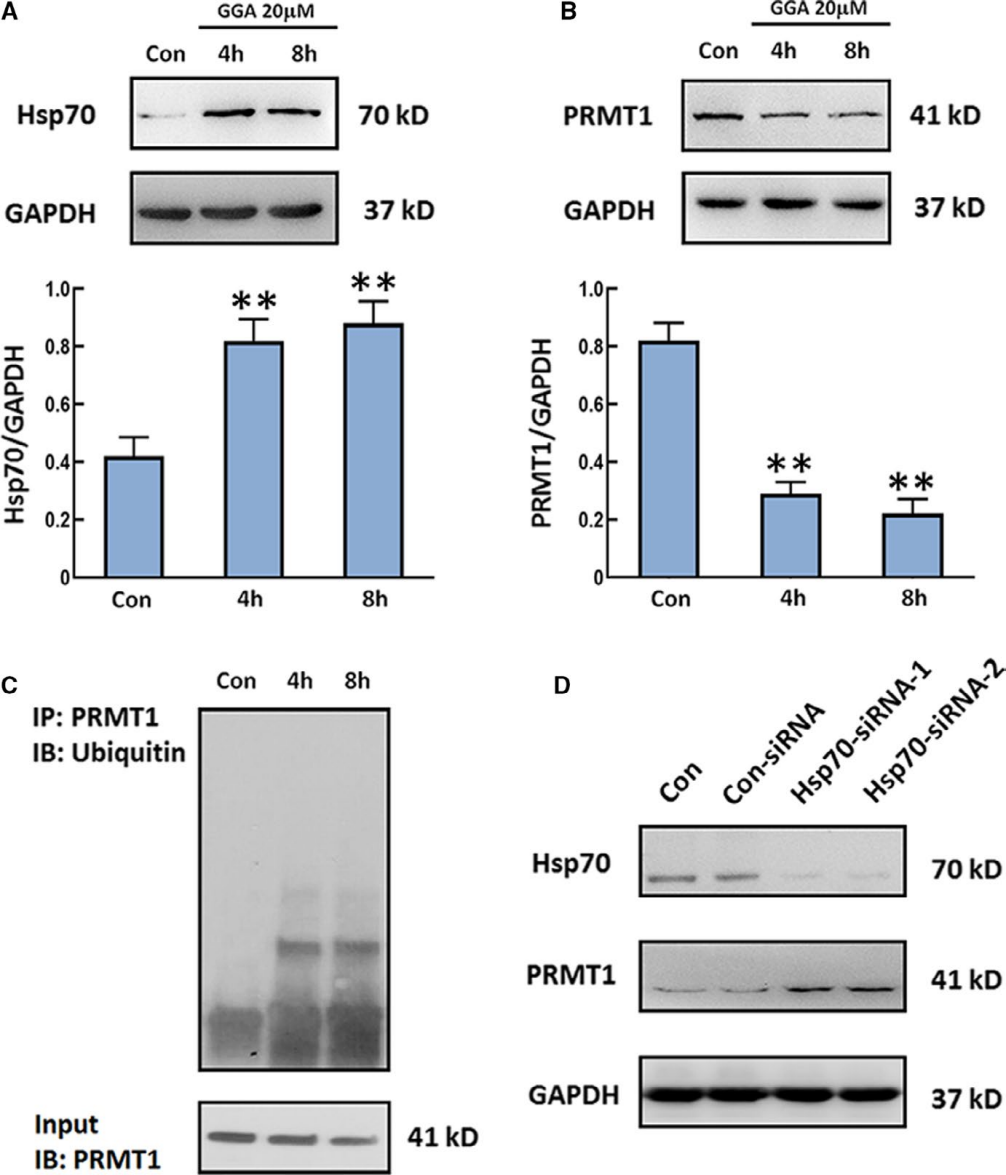
The influence of GGA on Hsp70 and PRMT1 levels and PRMT1 ubiquitination. U‐2 OS cells were treated with 20 µM GGA for 4 and 8 h. A and B, The expression of Hsp70 and PRMT1 was detected by Western blot. Quantification of the relative Hsp70 and PRMT1 levels (below). C, Ubiquitination of PRMT1 in U‐2 OS cells was assayed after MG132 treatment for 2 h by immunoprecipitation with anti‐PRMT1 antibody and immunoblotting with an antibody against ubiquitin. D, The expression of Hsp70 and PRMT1 was detected in U‐2 OS cells with/without Hsp70 siRNA transfection. n = 3. **P < 0.01 vs the Con group

Previous studies have reported that GGA acts as an inducer of Hsp70, which interacts with CHIP to promote protein degradation by polyubiquitination.^6^, ^14^ We have confirmed that CHIP could decrease PRMT1 level in HEK293 cells via the ubiquitination‐proteasome pathway.^14^ Here, we detected the levels of Hsp70 and PRMT1 in GGA‐stimulated U‐2 OS cells. The expressions of Hsp70 were obviously higher in U‐2 OS cells after GGA stimulation for 4 and 8 hours (Figure 3A). Nevertheless, the opposite trend was observed for PRMT1 expressions in cells. Compared with control, the expression of PRMT1 in GGA‐treated cells was markedly decreased (Figure 3B). We also observed polyubiquitination of PRMT1 in U‐2 OS cells treated with GGA for 4 and 8 hours. Cells were treated with proteasome inhibitor MG132 before harvesting to inhibit protein degradation. The ubiquitination of PRMT1 in different cell populations was revealed by immunoprecipitation with anti‐PRMT1 antibody and immunoblotting with anti‐UB antibody. Protein from cells without GGA treatment was used as a control. The results showed that the polyubiquitination of PRMT1 was obviously increased in cells after GGA stimulation for 4 and 8 hours (Figure 3C). To explore the direct relationship between Hsp70 and PRMT1, we examined the levels of PRMT1 in U‐2 OS cells treated with the siRNA against Hsp70. The data showed knockdown of Hsp70 increased the levels of PRMT1 in U‐2 OS cells (Figure 3D), suggesting that PRMT1 was negatively correlated with Hsp70 expression in U‐2 OS cells. These results indicate that GGA down‐regulates the level of PRMT1 through inducing Hsp70 expression in U‐2 OS cells.

### 3.4 The effect of GGA on PRMT1 degradation was achieved by CHIP‐mediated ubiquitination modification

**FIGURE 4:**
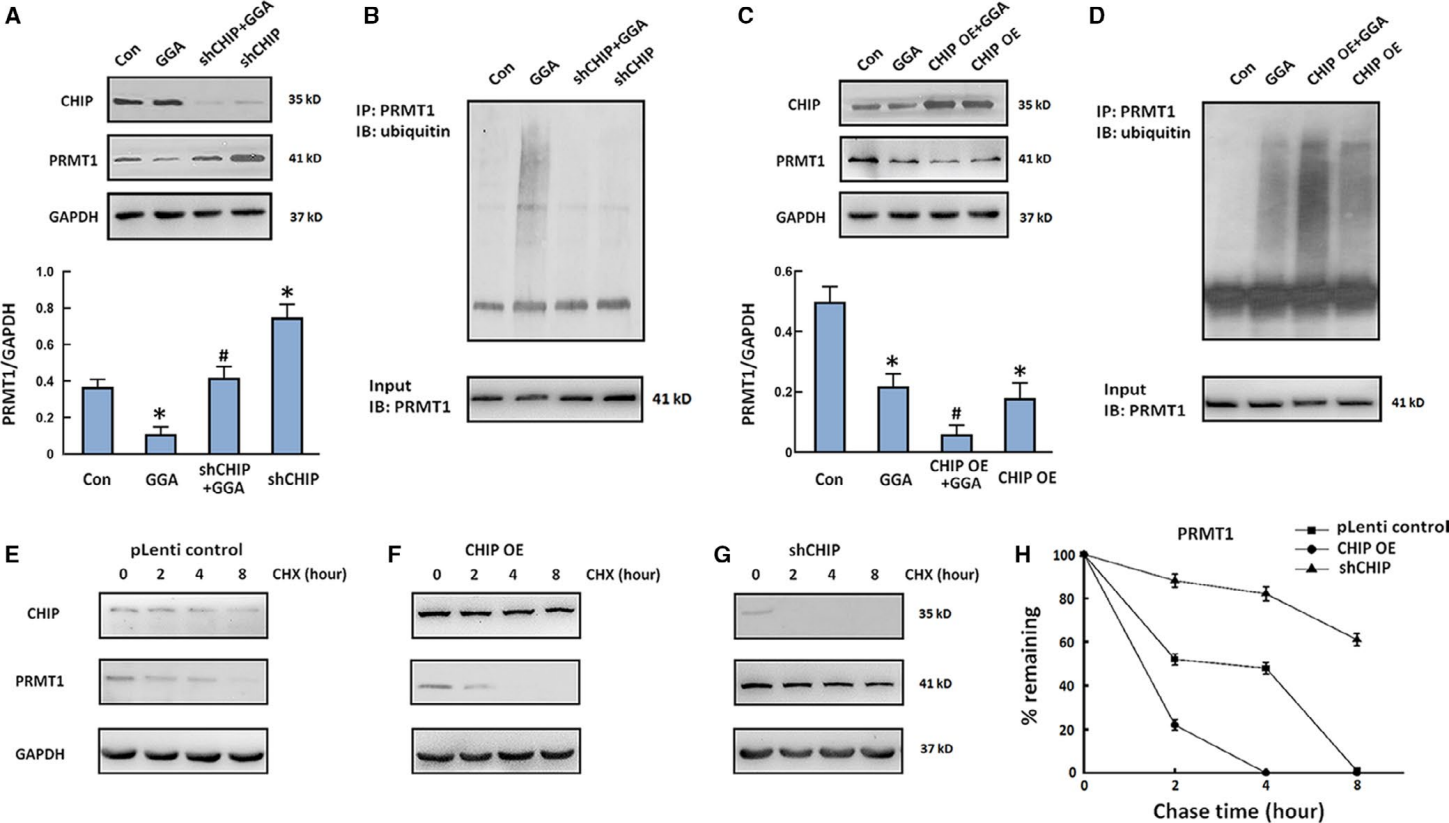
Effect of CHIP expression on the ubiquitination and stability of PRMT1. A and C, U‐2 OS cells transfected with shCHIP (A) or pLenti‐CHIP (C) plasmids were stimulated with GGA for 4 h. Western blot detection of PRMT1 and CHIP expression. Quantification of the relative PRMT1 level (below). B and D, Cell lysates were harvested after MG132 treatment for 2 h and immunoprecipitated with anti‐PRMT1 antibody followed by Western blot with anti‐ubiquitin antibody. E‐G, U‐2 OS cells were transfected with pLenti control (E), pLenti‐CHIP (F) or shCHIP (G) and then treated with cycloheximide (CHX, 100 µg/ml). Cell extracts were collected at 0, 2, 4 and 8 h after incubation with CHX followed by Western blot. H, Quantitative analysis of the CHIP and PRMT1 levels in the CHX chase experiment. n = 3. *P < 0.05 vs the Con group. ^#^ *P* < 0.05 vs the GGA group

To investigate whether GGA‐induced ubiquitination and degradation of PRMT1 are dependent on CHIP, we transfected U‐2 OS cells with short hairpin RNA against CHIP (shCHIP) and pLenti‐CHIP plasmids to knockdown or overexpress CHIP, respectively. The results showed that the expression of PRMT1 in GGA‐treated cells was restored by CHIP knockdown (Figure 4A). The GGA‐induced ubiquitination of PRMT1 was much weaker in U‐2 OS cells transfected with shCHIP than that in cells transfected with an empty vector (Figure 4B). Furthermore, U‐2 OS cells overexpressing CHIP exhibited PRMT1 degradation and more intense polyubiquitination of PRMT1 than those in parental cells with or without GGA treatment (Figure 4C and D). We also measured the half‐lives of the target proteins with the cycloheximide (CHX) chase assay. The cells were treated with CHX to inhibit protein synthesis, and the PRMT1 levels in the cell following CHX treatment for 0, 2, 4 and 8 hours were measured by immunoblotting with anti‐PRMT1 antibody. We found that the turnover of PRMT1 was markedly faster in cells overexpressing CHIP than that in control cells transfected with vector (Figure 4E and F). On the other hand, inhibiting endogenous CHIP expression in U‐2 OS cells with shRNA stabilized PRMT1 level (Figure 4G and H). These results confirm that the effect of GGA on PRMT1 degradation and ubiquitination is dependent on CHIP expression in U‐2 OS cells.

### 3.5 PRMT1 promoted the methylation and activation of STAT3 that suppressed the transcription of FAS

**FIGURE 5:**
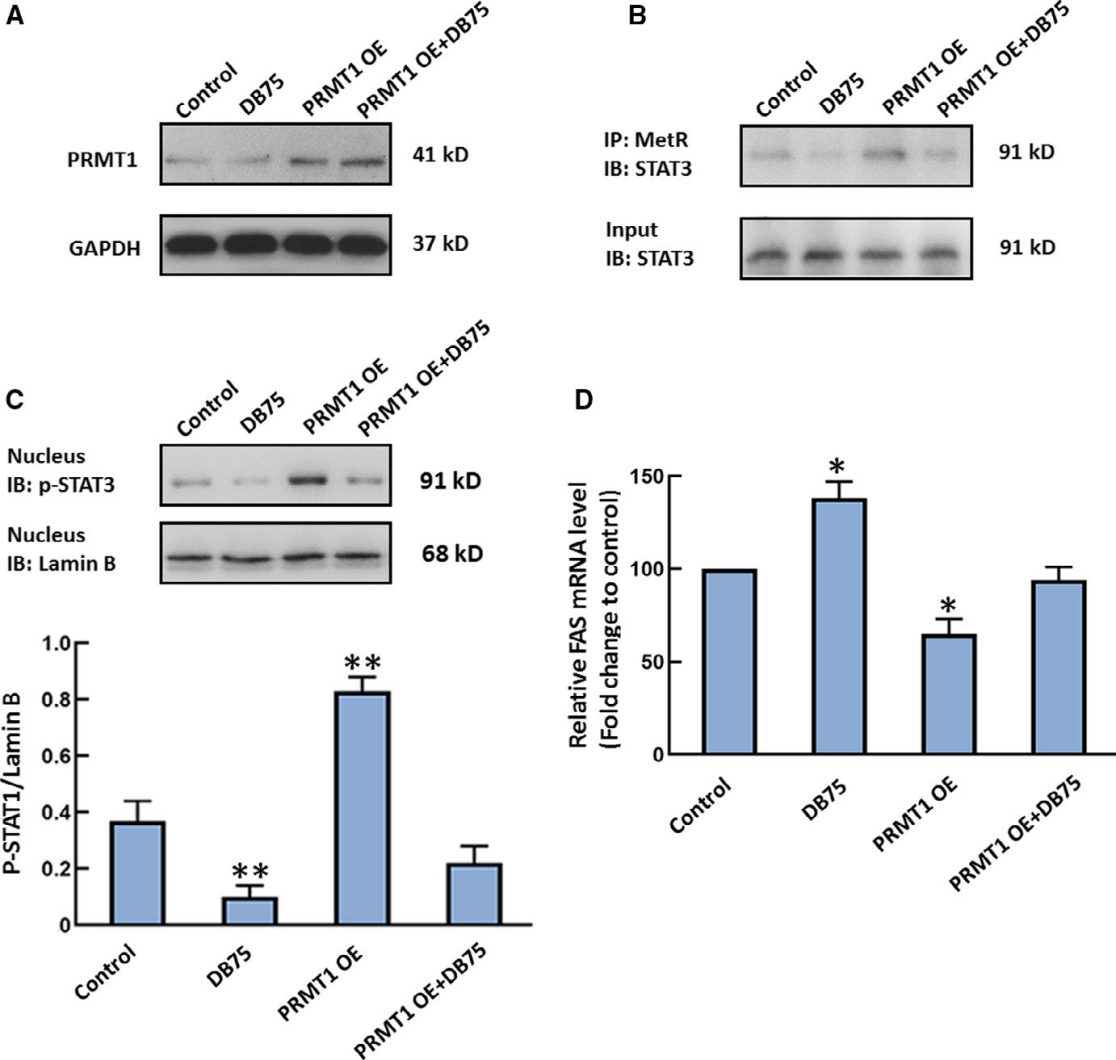
The effect of PRMT1 on the methylation of STAT3 and FAS transcription. U‐2 OS cells transfected with pLenti control or pLenti‐PRMT1 plasmids were administrated with 5 µM furamidine dihydrochloride (DB75), a PRMT1 inhibitor, for 8 h. A, The expression of PRMT1 was detected by Western blot. B, Methylation of STAT3 was tested by immunoprecipitation with anti‐mono and dimethyl‐arginine antibody (MetR) and immunoblotting with an antibody against STAT3. C, The phosphorylation of STAT3 was tested in nucleus by Western blot. D, The mRNA level of FAS was detected by qPCR assay. The mRNA analysis result is expressed as the fold increase over control. n = 3. *P < 0.05 vs the Con group. **P < 0.01 vs the Con group

Mizuki et al have reported that PRMT1 methylated arginine residues of STAT3 to regulate its activity positively in neural stem/precursor cells.^19^ Moreover, transcription of FAS is negatively regulated by activation of STAT3.^20^ Accordingly, we presumed that PRMT1 methylated STAT3 and enhanced STAT3 activity, which inhibited the transcription of FAS in U‐2 OS cells. We employed pLenti‐PRMT1 plasmid and a selective PRMT1 inhibitor, furamidine dihydrochloride (DB75), to detect whether PRMT1 plays a role in regulating STAT3 methylation and activation in U‐2 OS cells. Cell lysates derived from DB75‐treated cells and cells transfected with pLenti‐PRMT1 were collected to detect methylation of STAT3 by immunoprecipitation using anti‐mono and dimethyl‐arginine antibody. The isolated proteins were then analysed for the presence of STAT3 by immunoblotting. As shown in Figure 5A and B, the higher expression of PRMT1 was observed in U‐2 OS cells with pLenti‐PRMT1 transfection. The STAT3 was methylated even in untreated cells. However, the level of STAT3 immunoprecipitated was decreased in cells treated with DB75, whereas the level of STAT3 immunoprecipitated was increased in U‐2 cells with PRMT1 overexpression. The level of STAT3 phosphorylation in nucleus was also detected by Western blot. DB75 reduced the phosphorylation level of STAT3 in the nucleus. Overexpression of PRMT1 promoted phosphorylated STAT3 entry into nucleus of U‐2 OS cells (Figure 5C). To confirm whether the transcriptional level of FAS was affected by PRMT1, we performed qPCR analysis for level of FAS mRNA. The data showed that DB75 increased the mRNA level of FAS, which was reversed by exogenous PRMT1 (Figure 5D). These data indicated that methylation and activity of STAT3 are induced by PRMT1, resulting in the inhibition of FAS transcription.

### 3.6 Overexpression of PRMT1 suppressed GGA‐induced U‐2 OS cell apoptosis by inhibiting the FAS‐triggered apoptotic pathway

**FIGURE 6:**
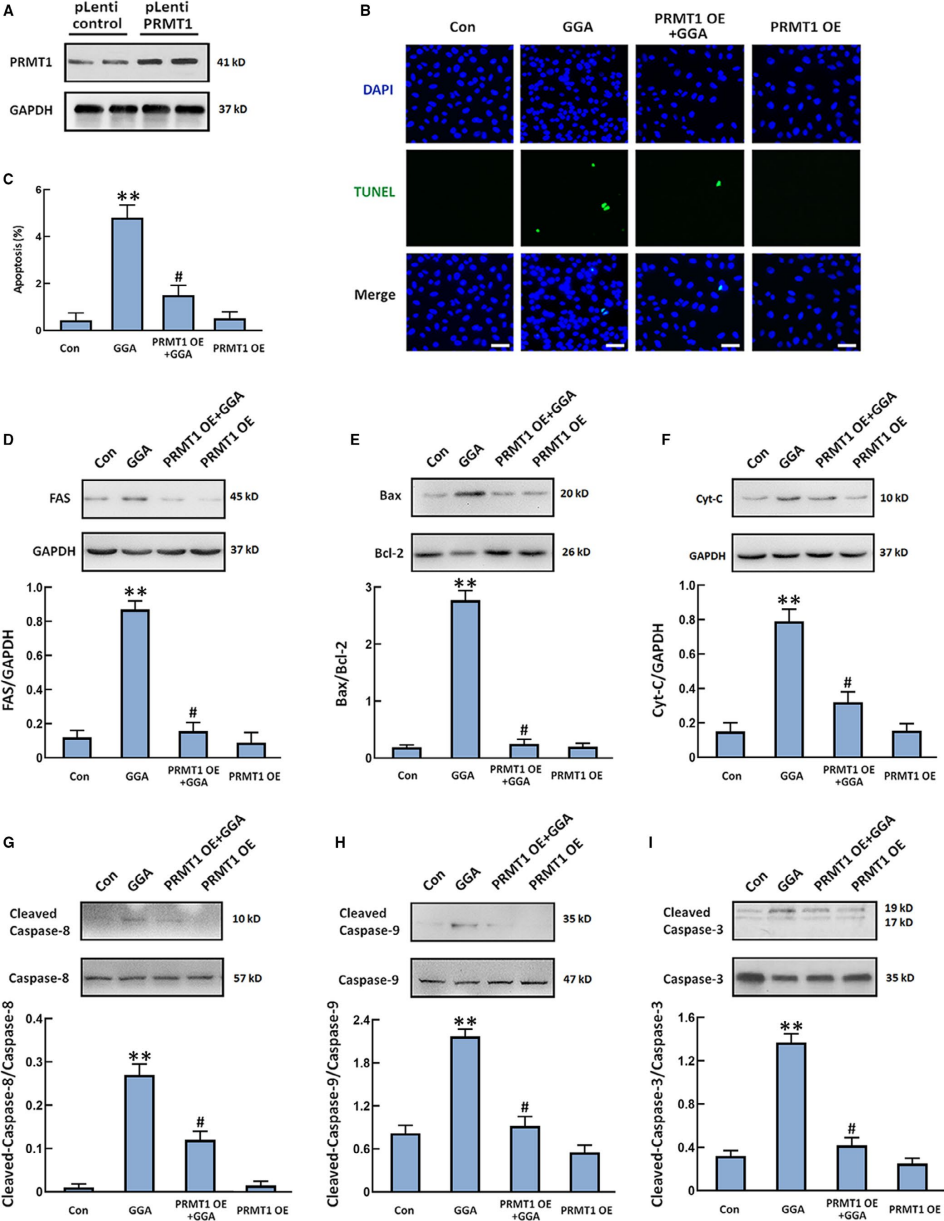
Overexpression of PRMT1 suppresses GGA‐induced U‐2 OS cell apoptosis by inhibiting the FAS‐regulated apoptotic pathway. U‐2 OS cells were transfected with pLenti control or pLenti‐PRMT1 plasmids and treated with 20 µM GGA. A, Western blot detection of PRMT1 expression in U‐2 OS cells. B, Representative photomicrographs of the TUNEL assay (green). Total nuclear staining with DAPI (blue). Scale bar =100 µm. C, The numbers of apoptotic cells were counted in five randomly selected fields for each sample based on the TUNEL images. D‐I, FAS expression (D), the Bax/Bcl‐2 ratio (E), Cyt c release (F), cleaved caspase‐3/caspase‐3 expression (G), cleaved caspase‐8/caspase‐8 expression (H) and cleaved caspase‐9/caspase‐9 expression (I) were detected by Western blot. Quantification of the relative protein levels in the panels under the images. n = 3. **P < 0.01 vs the control group. ^#^ *P* < 0.05 vs the GGA group

The role of PRMT1 in translation regulation has been recognized.^21^ This study next sought to determine whether high expression of PRMT1 inhibits GGA‐induced U‐2 OS cell apoptosis. We overexpressed PRMT1 in U‐2 OS cells by pLenti‐PRMT1 transfection and then measured cell apoptosis and apoptotic protein levels after GGA stimulation. As shown in Figure 6A‐C, Western blot of the cell lysates with an anti‐PRMT1 antibody revealed higher PRMT1 expression in transfected U‐2 OS cells. In parallel, overexpression of PRMT1 reduced apoptotic cell proportion after GGA treatment for 24 hours. We further investigated the effect of PRMT1 on GGA‐induced activation of apoptosis‐related proteins in U‐2 OS cells. The results showed that FAS expression, the Bax/Bcl‐2 ratio, cytochrome c (Cyt c) release and caspase‐3, caspase‐8 and caspase‐9 activity were markedly elevated when 20 μM GGA was administered to U‐2 OS cells for 12 hours. Overexpression of PRMT1 decreased GGA‐induced high levels of FAS expression, the Bax/Bcl‐2 ratio, Cyt c release and caspase‐3, caspase‐8 and caspase‐9 activation in U‐2 OS cells (Figure 6D‐I). These observations indicate that PRMT1 restrains GGA‐mediated U‐2 OS cell apoptosis by inhibiting the FAS‐triggered apoptotic pathway.

### 3.7 PRMT1 was up‐regulated at the protein level in OS tissues of patients

**FIGURE 7:**
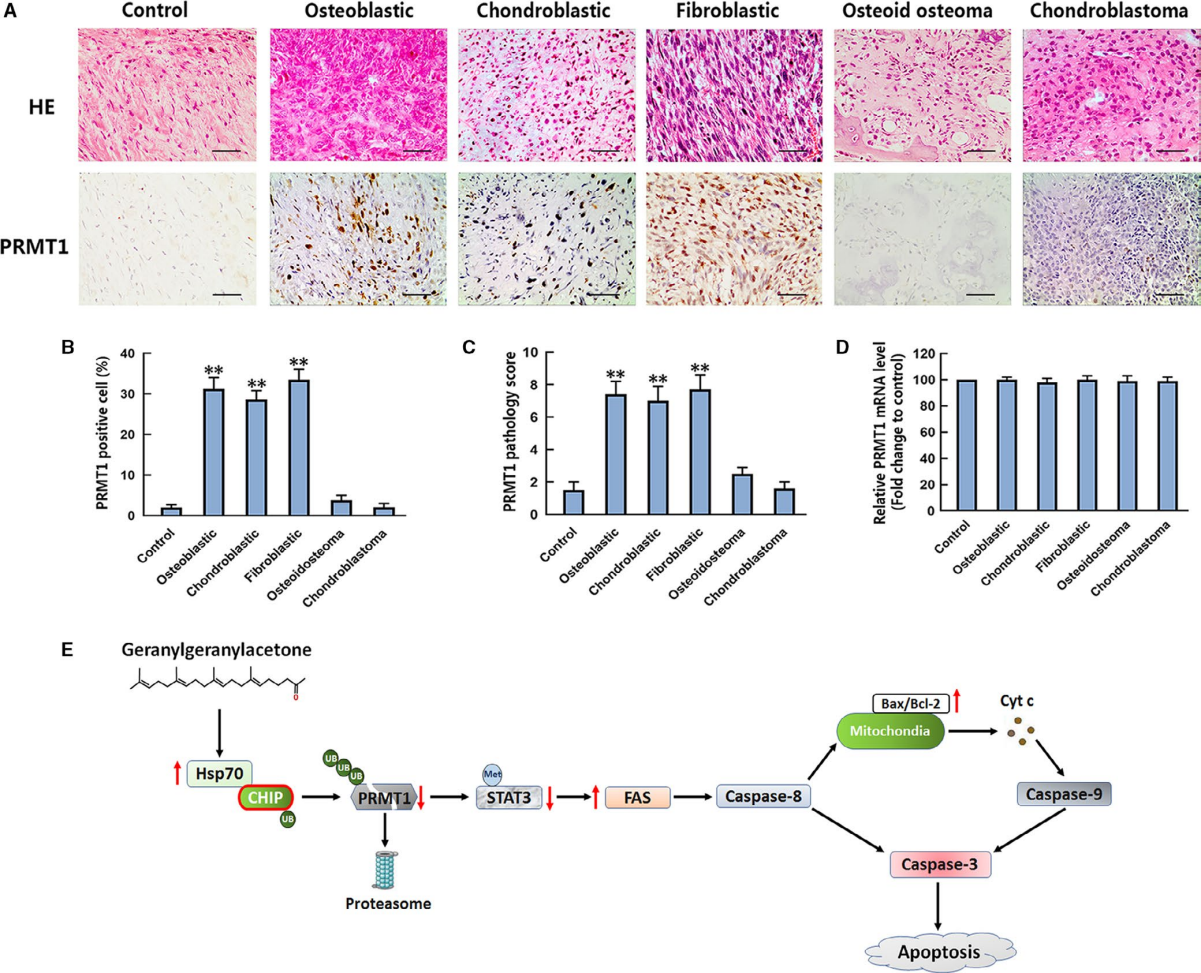
Evaluation of PRMT1 protein and mRNA levels in OS and two benign bone tumours. A, Representative 20× images of H&E staining (upper row) and anti‐PRMT1 antibody staining (bottom row) of control, osteoblastic, chondroblastic, fibroblastic OS, osteoid osteoma and chondroblastoma. Scale bar =200 µm. B, The percentages of PRMT1‐positive cells were calculated based on five randomly selected fields in the stained control, OS and benign bone tumour tissues. C, The pathology scores of PRMT1‐positive staining in the control and bone tumour tissues. The means of the scores of five randomly selected fields of tissues were used for plotting. D, The mRNA level of PRMT1 in control and tumours tissues was detected by qPCR assay. The mRNA analysis result is expressed as the fold increase over control. n = 8‐22. E, Signalling mechanisms of GGA‐induced U‐2 OS cell apoptosis. UB, ubiquitination. Met, methylation. **P < 0.01 vs the control group

Aberrant expression of PRMT1 has been reported in several malignancies.^16^ We detected the expression of PRMT1 via immunohistochemical staining of clinical samples from three major subtypes of conventional OS (osteoblastic, chondroblastic and fibroblastic) and two types of benign bone tumours (osteoid osteoma and chondroblastoma). The adjacent normal tissues were set as the control group. As shown in Figure 7A, PRMT1‐positive staining was predominantly observed in osteoblastic, chondroblastic and fibroblastic OS samples. The proportion of PRMT1‐positive cells was much lower in control than that in OS tissues. There is no significant difference in PRMT1 expressions between control and benign tumour tissues. We also counted the PRMT1‐positive cells in the OS and benign tumour tissues and calculated the pathological scores associated with PRMT1 expression. Our analysis confirmed the increase in the number of PRMT1‐positive cells and the corresponding pathological scores of the OS tissues (Figure 7B and C). Moreover, the mRNA levels of PRMT1 in bone tumour and control tissues were measured by qPCR. The data showed that, at the mRNA level, there was no marked difference of PRMT1 among the groups (Figure 7D). These results ruled out the possibility of PRMT1 transcriptional change in OS tissues. We also examined the expression of Hsp70 in all samples that were selected out. There was also no significant difference in the Hsp70‐positive staining among the groups (Figure S1).

## 4 DISCUSSION

GGA is widely involved in cell homeostasis and related processes, including cell growth, differentiation, proliferation and apoptosis.^22^ A few studies have demonstrated that GGA decreased cell viability and proliferation, as well as induced cell apoptosis in multiple types of cancer cells, including breast cancer, colon cancer and melanoma cells.^7^, ^8^, ^9^ However, the effects of GGA on human OS have not been investigated. In the present study, the viability and proliferation of cells were suppressed in a dose‐dependent manner after 24 hours of GGA treatment. Furthermore, GGA markedly increased FAS expression and activated caspase‐3, caspase‐8 and caspase‐9 along with the obvious cell death, as indicated by TUNEL staining in U‐2 OS cells.

GGA has been identified as a non‐invasive Hsp70 inducer, and the cooperative activity of Hsp70 and CHIP is involved in the quality control of multiple client proteins.^23^ In our previous study, we found that CHIP can promote PRMT1 ubiquitination and degradation in HEK293 cells. PRMT1 is a histone arginine methyltransferase that mainly mono‐ and demethylates histone H4 arginine 3, which is an activation site for gene expression.^25^, ^26^ In colon cancer, PRMT1 is involved in epidermal growth factor receptor methylation during the resistance to cetuximab treatment. In addition, the down‐regulation of PRMT1 induces the apoptosis in human colon cancer cells.^27^, ^28^, ^29^ Therefore, we hypothesized that cell apoptosis induced by GGA might be closely associated with the degradation of PRMT1, which was mediated by the Hsp70‐CHIP proteasome pathway. In this study, increased expression of Hsp70 in U‐2 OS cells was observed after GGA stimulation for four and eight hours. In stark contrast, the protein expression of PRMT1 was inhibited in GGA‐treated cells. Furthermore, transient expression and siRNA knockdown of CHIP were performed in U‐2 OS cells. Accordingly, overexpression of CHIP enhanced PRMT1 degradation and MG132‐induced accumulation of ubiquitylated PRMT1 in U‐2 OS cells after GGA stimulation. We also found that either CHIP or Hsp70 knockdown suppresses PRMT1 degradation and induces its accumulation. The above results further prove that the ubiquitination and degradation of PRMT1 are regulated by CHIP and Hsp70 in U‐2 OS cells.

Although the cytotoxic effect of GGA in cancer cells has been reported, the exact molecular mechanism of GGA‐induced apoptosis has not been fully explored. How GGA activates caspase‐3 is still a mystery. The classical pathway of p53‐dependent apoptosis exploits the Bax‐mediated release of Cyt c and AIF, which are actively involved in caspase activation and protein or DNA degradation.^29^ Besides this, another apoptotic pathway is triggered by extracellular signals that activate FAS. The FAS‐associated death domain protein bridges FAS with the downstream effector caspase‐8, forming the death‐inducing signalling complex that ultimately results in Bax increase and the release of Cyt c from mitochondria. This sequence of events induces the activation of caspase‐9 and caspase‐3, which leads to cell apoptosis.^31^, ^32^ In the present study, we observed that 20 µM GGA treatment did not obviously affect p53 content in U‐2 OS cells. In contrast, GGA markedly increased the level of FAS, but this effect was inhibited by PRMT1 overexpression. Other studies have reported that STAT3 is a methylation target of PRMT1 in neural stem/precursor cells. Knockdown of STAT3 expression by siRNA induced FAS‐mediated apoptosis in vitro and in vivo in breast cancer.^20^, ^33^ Our study demonstrated that PRMT1 promoted methylation and activation of STAT3, leading to inhibition of FAS transcription in U‐2 OS cells. Furthermore, overexpression of PRMT1 apparently reversed GGA‐induced increases in the Bax/Bcl‐2 ratio, Cyt c release, activation of caspase‐3, caspase‐8 and caspase‐9 and cell apoptosis. Therefore, the effect of GGA on the inhibition of PRMT1 expression may contribute to the suppression of U‐2 OS cell progression through the up‐regulation of FAS.

OS is the most common primary malignant bone tumour in adolescents. Three major subtypes of the conventional OS are recognized, reflecting the predominant phenotype of the tumour matrix: osteoblastic, chondroblastic and fibroblastic. Through our research findings, we speculated that the expression of PRMT1 could be changed in OS tissue. Therefore, we detected the expression of PRMT1 in both conventional OS and two types of benign bone tumours, osteoid osteoma and chondroblastoma, by immunohistochemistry. Our data demonstrate that the proportion of cells with positive PRMT1 expression was obviously higher in OS patient samples than that in control and benign bone tumours. The mRNA analysis ruled out the possibility of PRMT1 transcriptional change in OS tissue. The data indicated that the increased expression of PRMT1 in OS was associated with the regulation of post–translational modifications. The expressions of Hsp70 were not different among the various tissue samples, which means that, in addition to Hsp70‐mediated pathway, there may be other mechanisms that regulate the expression of PRMT1 in the OS tissues. PRMT1 may serve as a potential diagnostic marker and new therapeutic target for patients with OS.

Overall, the present study demonstrated that the suppressive effect of GGA on human OS cells is mediated by accelerating the ubiquitination and degradation of PRMT1 through the Hsp70‐CHIP proteasome pathway. The degradation of PRMT1 in U‐2 OS cells induces the inhibition of STAT3 methylation and activation of the FAS‐triggered apoptotic pathway, resulting in cell apoptosis (Figure 7E). In addition, high expression of PRMT1 is observed in the three major subtypes of OS but not in osteoid osteoma and chondroblastoma. Our study found a novel effect of GGA on human OS cells. However, more studies are needed to dissect further the role of GGA in OS animal model for future clinical trials.

## CONFLICT OF INTEREST

The authors confirm that there are no conflicts of interest.

## AUTHOR CONTRIBUTION

**Lucen Jiang:** Data curation (equal); Formal analysis (equal); Methodology (equal); Writing‐original draft (equal). **Jia Liao:** Data curation (equal); Formal analysis (equal); Software (equal). **jianghuan liu:** Data curation (equal); Methodology (equal); Validation (equal). **qingzhu wei:** Project administration (equal); Resources (equal); Supervision (equal). **yiyang wang:** Conceptualization (lead); Funding acquisition (lead); Project administration (lead); Resources (equal); Supervision (equal); Writing‐review & editing (equal).

## Supporting information

Supplementary Material

## Data Availability

The data that support the findings of this study are openly available on request from the corresponding author.
