# Supplementary material for: Geranylgeranylacetone promotes human osteosarcoma cell apoptosis by inducing the degradation of PRMT1 through the E3 ubiquitin ligase CHIP
Source: J Cell Mol Med. 2021 Jun 21;25(16):7961–72. doi: 10.1111/jcmm.16725 (PMC8358878; doi:10.1111/jcmm.16725)
Supplement: Supplementary file 1 — Supplementary Material [file JCMM-25-7961-s001.docx]

**Supplementary**

**Figure S1**

**
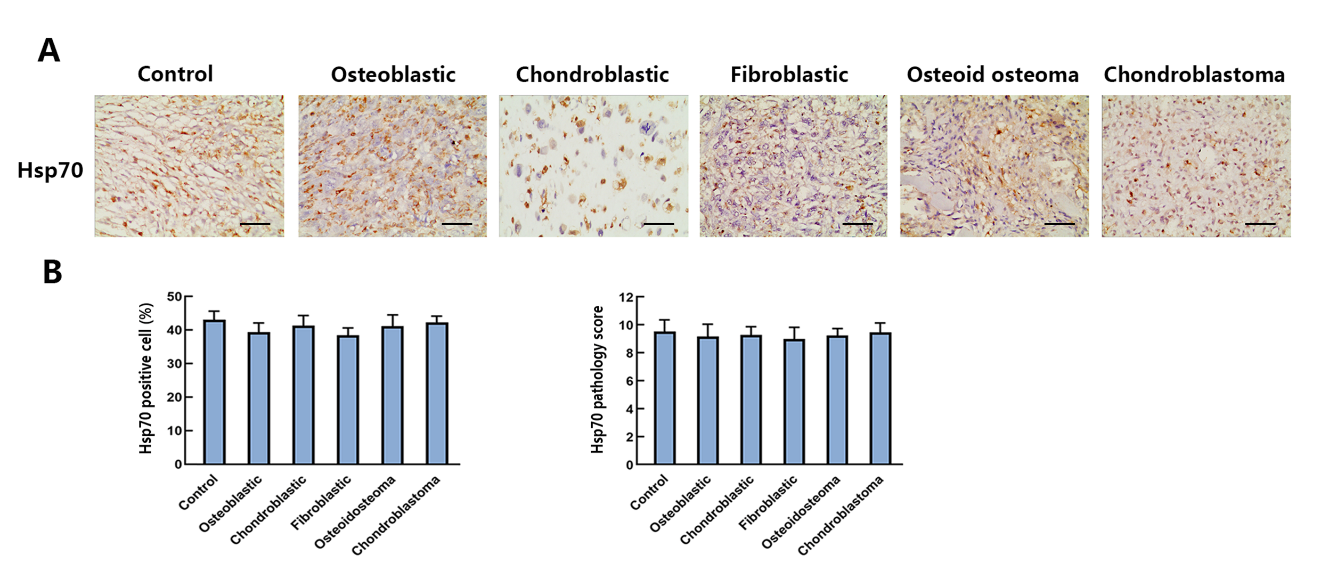
**

**Fig. S1** **Evaluation of Hsp70 expression in bone tumors tissues.** A, Representative 20× images of anti-Hsp70 antibody staining of control, osteoblastic, chondroblastic, fibroblastic OS, osteoid osteoma and chondroblastoma. Scale bar = 200 μm. B, The percentage and pathology score of PRMT1-positive cells were calculated based on five randomly selected fields in the stained control, OS and benign bone tumor tissues. The means of the scores of five randomly selected fields of tissues were used for plotting. n=8-22.
